# Development of Fluorescent FRET Probes for “Off-On” Detection of L-Cysteine Based on Gold Nanoparticles and Porous Silicon Nanoparticles in Ethanol Solution

**DOI:** 10.3390/s17030520

**Published:** 2017-03-05

**Authors:** Hongyan Zhang, Zhenhong Jia

**Affiliations:** 1 School of Physical Science and Technology, Xinjiang University, Urumqi 830046, China; zhanghongyanxj@163.com; 2 College of Information Science and Engineering, Xinjiang University, Urumqi 830046, China

**Keywords:** “off-on” fluorescence biosensor, porous SiNPs, ethanol solution, AuNPs, L-Cysteine

## Abstract

A new type of fluorescence “off-on” probe was designed for L-Cysteine (L-Cys) based on the fluorescence resonance energy transfer (FRET) between negatively charged amino-capped porous silicon nanoparticles (SiNPs) and positively charged citrate-stabilized Au nanoparticles (AuNPs). In this proposed FRET immunosensor, novel water-soluble amino-conjugated porous SiNPs in ethanol with excellent photoluminescence properties act as the energy donor. Excellent quenching efficiency between SiNPs-ethanol and citrate-stabilized AuNPs by electrostatic interaction via FRET provides an ideal “off-state” (turn-off). The addition of L-Cys leads to releasing the adsorbed AuNPs from the surface of SiNPs and hence the fluorescence emission of SiNPs-ethanol is restored (turn-on), which means the coordination ability of the thiols with AuNPs is stronger than that of the electrostatic interaction. The fluorescence intensity of SiNPs-AuNPs in ethanol is sensitive to L-Cys, and such a restored fluorescence is proportional to the concentration of L-Cys. The method will broadly benefit the development of a new thiol biosensor based on nanostructured porous materials, and the proposed procedure is also expected to develop a variety of functional nanoparticles to form other novel kinds of nanosensors.

## 1. Introduction

In recent years, the study of biologically thiol-contained amino acids has drawn great attention due to their crucial roles in a wide range of biological processes. L-Cysteine (L-Cys) as a small-molecular-weight biological thiol-contained amino acid, which provides an important role in several biological processes and is essential for maintaining the appropriate redox status of proteins, cells or organisms [1,2]. It is also an active site in the catalytic function of certain enzymes called cysteine proteases and in many other peptides and proteins, and it can react with certain proteins by splitting the disulfide bonds. Altered levels of L-Cys have been associated with many diseases, such as slow growth, liver damage, hair depigmentation, skin lesions, loss of muscle and fat, Alzheimer’s disease and Parkinson’s disease, etc. [3,4]. In view of the significance of L-Cys, it is necessary to develop a simple and rapid method to detect and determine the concentration of L-Cys in environmental and biological samples with high sensitivity and selectivity. Many analytical techniques are described in the literature for the determination of L-Cys, such as chemiluminescence, chromatography, spectrophotometry, electrochemical methods, spectrofluorimetry, flow injection analysis and so on [5,6,7]. Though the above methods are widely used, they all have their limits, such as high cost or robust sample handling. Furthermore, most of those methods are based on redox chemistry, derivatization with chromophores, and also involve the use of high temperature conditions and hazardous reagents. The development and application of an effective analytical method for routine monitoring of L-Cys is therefore necessary.

The spectrofluorimetry method is considered the best method to satisfy high selectivity and sensitivity with easy measurement. Most fluorescence sensors are constructed by fluorescence resonance energy transfer (FRET) sensors which are designed as fluorescence “off-on” probes, or they exhibit a signal output of fluorescence emission quenching [8,9,10]. In particular, FRET is a distance-dependent energy transfer technique that has also been utilized for L-Cys determination. The FRET methods for L-Cys recognition mostly based on thiol can be bound on the surface of noble metal nanoparticles via the metal-sulfur bond, where the interaction is strong enough to immobilize the thiol groups on the surface of nanoparticles. Gold nanoparticles (AuNPs) have been widely utilized as amionthiol sensors because of their surface plasmon resonance and strong affinity to thiols. In the FRET system, AuNPs are able to quench the fluorescence of fluorescent materials when a spectral overlap between the absorption spectrum of AuNPs and the emission spectrum of fluorescent materials exists [11,12,13]. Thus, the construction of fluorescent materials for L-Cys sensing is designed to be “off-on” [10,14,15,16,17]. However, the fluorescence spectrum band of traditional fluorescent materials including rhodamine B, gold nanoclusters (AuNCs) or silica nanoparticles is relatively broad and asymmetric [18,19,20], and the effective overlap of the absorbance spectrum of AuNPs or the fluorescence spectrum band of traditional fluorophores is narrow, which affects the detection sensitivity of the immunosensor. There is a wide overlap of the absorbance spectrum of AuNPs and the fluorescence emission wavelength of fluorescent quantum dots (QDs), but the method is limited by a need for well-trained professionals and the high cost of QDs. Furthermore, some disadvantages are still retained such as cytotoxicity as a result of the release of heavy metal ions, strong dependence of the photoluminescence (PL) on the surface states, as well as chemical and colloidal instabilities of the QDs in harsh environments [14,21,22]. Silicon nanoparticles (SiNPs) are recognized as one type of important semiconductor nanomaterial to overcome these drawbacks. There are some unique properties of silicon which are noticeable when its structure is reduced to the nanodimension [23], such as emitting in the infrared window, an adjusted emission wavelength, different hydrophilicity or hydrophobicity under different surface modifications, good biocompatibility and being a low-cost material capable of being used at room temperature. Thus SiNPs are used in a large number of applications in sensing, optoelectronics, micromachining, biotechnology, wafer technology, etc. [23,24]. In order to explore the extensive analytical applications of SiNPs as chemosensors and immunosensors, one desirable topic is how to develop simple, low-cost and selective fluorophores.

In our experiments, we used novel water-soluble amino-capped porous SiNPs in ethanol as the donor to obtain excellent photoluminescence properties. Based on the advantages of AuNPs and SiNPs, a new type of fluorescence “off-on” probe for L-Cys was developed. Such a FRET immunosensor provides an ideal “off-state” by an excellent quenching efficiency between SiNPs-ethanol and citrate-stabilized AuNPs via electrostatic interaction. Upon the addition of L-Cys to the SiNPs-AuNPs system, the fluorescence emission was restored, which means L-Cys can release the adsorbed AuNPs from the surface of SiNPs because of the stronger coordination ability of the thiols with AuNPs compared to that of the electrostatic interaction. This restored fluorescence intensity is proportional to the concentration of L-Cys. Due to this ability, porous SiNPs in ethanol solution as a donor can be used to detect L-Cys. To the best of our knowledge, this is the first report of SiNPs and AuNPs in ethanol to detect L-Cys. The results show that it is a valid model for establishing other thiol biosensors for the detection of L-Cys.

## 2. Materials and Methods

### 2.1. Chemicals and Reagents

Sodium citrate, glutathione (GSH), L-Cysteine (L-Cys) and 3-Aminopropyltriethoxysilane (APTES) were purchased from Aladdin Reagent Co., Ltd. (Shanghai, China). Chloroauric acid (HAuCl_4_·4H_2_O_2_, 48%–50% Au basis) was purchased from Macklin Co., Ltd. (Shanghai, China). Phosphate buffer saline pH 7.4 (0.01 M PBS buffer solution) was obtained from Sangon Biotech Co., Ltd. (Shanghai, China). All other reagents were analytical grade and used without further purification. Deionized water (DI) was used throughout the study.

### 2.2. Instruments

Fluorescence measurements were carried out on an F-4600 spectrophotometer (Hitachi, Tokyo, Japan) with the wavelength ranging from 200.00 nm to 900.00 nm. The spectra was obtained under the condition that photomultiplier tube (PMT) voltage was 700 V, excitation and emission slits was 5 nm and excitation wavelength was 350 nm. The high solution transmission electron microscopic (HR-TEM) images were obtained from a JEM-2100F transmission electron microscope (Hitachi, Tokyo, Japan). Absorption spectra were acquired on a Lambda 650 UV-vis spectrophotometer (PerkinElmer, Waltham, MA, USA) with the wavelength ranging from 200.00 to 1000.00 nm.

### 2.3. Sythesis of Citrate-Stabilized AuNPs

Aqueous dispersion of citrate-stabilized AuNPs were prepared using the citrate reduction method. Briefly, 20 mL mixture of 0.5 mM HAuCl_4_ and DI water was heated up to 90 °C under vigorous stirring for 5 min, and then 20 mL of 0.25 mM sodium citrate solution was quickly added into it. The color of the solution changed from faintly gray to claret-red. Under continuous stirring, reaction was allowed to continue for 30 min. Then, the solution was cooled down to room temperature and stored at 4 °C in a refrigerator. Citrate-stabilized AuNPs have negative charge.

### 2.4. Sythesis of Amino-Capped SiNPs

Amino-capped SiNPs were facilely prepared via simple one-pot electrochemical etching method, the as-prepared SiNPs can be passivated by hydrogen peroxide oxidation, and further aging was the use of silane-coupling reagent in the organic phase. Amino-capped SiNPs have positive charge and can be further functionalized with negative charged AuNPs.

In our experiment, porous silicon (PS) layer was prepared from p-type (100) silicon substrate with the resistivity of 0.02–0.03 Ω·cm and the thickness of 400 ± 10 µm. The Si substrate was electrochemically etched in a teflon electrochemical etch cell using a piece of Cu as the counter electrode, exposing an area of approximately 0.2 cm^2^ to a mixed solution with 49% aqueous hydrofluoric acid (HF) and ethanol (volume ratio 1:1). The electrolyte temperature was maintained at 15 °C for the duration of the reaction and the anodic constant current density is 55 mA/cm^2^ for 25 min. After being etched, the substrates were cleaned with deionized water (DI) and dried at room temperature in the air. The fresh PS substrate was put in 15 mL ethanol solution and ultrasonicated for 30 min until the PS layer was completely detached from the silicon substrate. The obtained mixture solution is composed of SiNPs and ethanol. Subsequently, 3 mL APTES and 300 μL H_2_O_2_ were added to the mixture solution, then the solution was ultrasonicated for 30 min and kept being shaken plentifully for 48 h. The product was purified by centrifugation at 10,000 rpm for 10 min, where the capped particles formed a solid mass at the bottom of centrifuge tube and the excess unreacted reagent was removed. The remaining particles were washed with ethanol and centrifuged again. This process was repeated three times before the particles were placed into 15 mL ethanol solution.

### 2.5. Procedures for Fluorescence Detection of L-Cys

First 1 mL amino-capped SiNPs and 2 mL citrate-stabilized AuNPs were added into a 10 mL centrifuge tube which was kept being shaken plentifully for 2 h at room temperature, then 1 mL different concentrations of L-Cys solutions were added into the obtained mixture system. At this stage, a typical FRET-based analysis of L-Cys was performed. Afterwards, the fluorescence emission spectra were recorded with the excitation of 350 nm. The calibration curve for L-Cys was established according to the fluorescence enhancement efficiency.

## 3. Results and Discussion

### 3.1. Characterization of AuNPs and SiNPs

**Figure 1:**
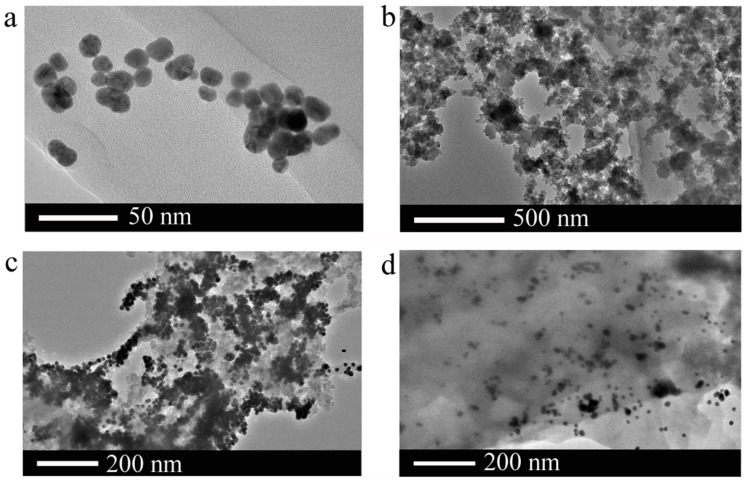
HR-TEM images of (**a**) AuNPs; (**b**) SiNPs; (**c**) AuNPs-SiNPs at the absence of L-Cys and (**d**) AuNPs-SiNPs in the presence of L-Cys.

The size and morphology of AuNPs and AuNPs-SiNPs were characterized by high-resolution transmission electron microscopy (HR-TEM) at different magnifications, as shown in Figure 1. In Figure 1a, the typical HR-TEM micrograph of AuNPs shows that uniform AuNPs are roughly spherical and the average diameter is about 14 nm. That means AuNPs synthesized by the citrate reduction method are stabilized by adsorbed negative citrate ions, and the electrostatic repulsion is maintained with a good dispersion of the AuNPs. In Figure 1b, the typical HR-TEM microscopy of SiNPs is exhibited, where the sponge-like porous SiNPs have a pore dimension of approximately 20–40 nm and the pore distribution is random. It should be noted that the as-prepared SiNPs have a good dispersity in ethanol solution due to the abundant NH_2_-contained functional groups on the surface.

To study the mechanism of the modulation in the fluorescence recovery of the AuNPs-SiNPs composite in ethanol by L-Cys, the morphology and aggregation state of the AuNPs-SiNPs were characterized by HR-TEM either in the presence or in the absence of L-Cys. As shown in Figure 1c, the addition of AuNPs into SiNPs-ethanol solution did result in the aggregation of AuNPs on the surface of SiNPs, which confirmed that many AuNPs successfully adhered to the surface of SiNPs through the electrostatic interaction between negatively charged amino-capped SiNPs and positively charged citrate-stabilized AuNPs. However, Figure 1d shows that only a few AuNPs were attached to the SiNPs when AuNPs-SiNPs were in the presence of L-Cys, indicating that the interaction of L-Cys with AuNPs can reverse the process from the aggregation of AuNPs to a dispersion state. The sulfur atom in the free thiol group can provide electron pairs to Au atoms on the AuNP surface. This characteristic makes it easier for it to be adsorbed on the surface of Au and to have a strong binding preference toward AuNPs by forming an Au-S bond on the SiNP surface. Furthermore, the AuNPs are detached from the SiNPs in the presence of L-Cys. Therefore, an attempt has been made to determine the L-Cys by spectrofluorimetry.

### 3.2. Optical Characteristics of AuNPs and SiNPs-Ethanol

**Figure 2:**
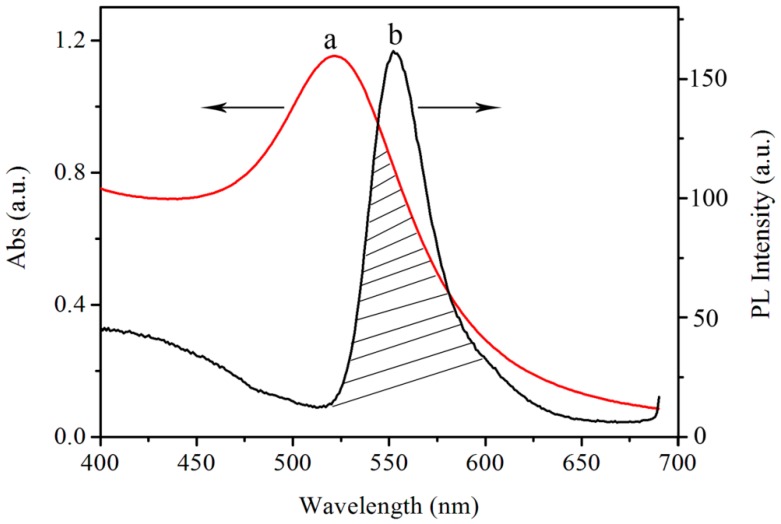
(**a**) Absorption spectrum of AuNPs and (**b**) fluorescence emission of SiNPs-ethanol.

The rate of energy transfer is highly dependent on many factors, such as the extent of the spectral overlap and the distance between the donor and acceptor molecules. Figure 2 shows the mechanism of the FRET detection of L-Cys using SiNPs-ethanol solution as the fluorescent donor and AuNPs as the fluorescent acceptor. For the UV-vis absorption spectra in Figure 2a, AuNPs exhibited a wide characteristic absorption spectrum with a maximum absorption at 523 nm. Figure 2b shows that the maximum fluorescence emission wavelength of the fluorescence SiNPs in ethanol solution is around 550 nm. It can also be seen that the fluorescence spectrum band is relatively narrow and symmetric, and is just near the maximum plasmon absorption of AuNPs. It is obvious that the spectrum overlap between the emission spectrum of SiNPs in ethanol and the absorption spectrum of AuNPs ensures the feasibility of this FRET biosensor.

**Figure 3:**
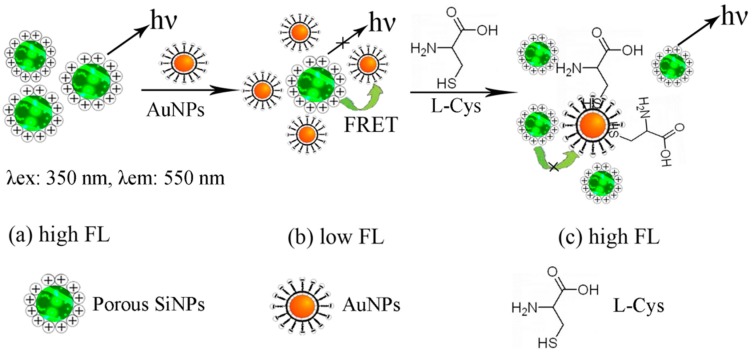
Schematic illustration for the detection of L-Cys based on the FRET between SiNPs-aqueous and AuNPs.

Figure 3 shows the principle of the “off-on” detection of L-Cys using SiNPs-ethanol solution as a fluorescent probe. Initially, the porous SiNPs in ethanol showed a strong fluorescence centered at around 550 nm when the excitation wavelength was 350 nm (Figure 3a). Upon the addition of AuNPs to the SiNPs-ethanol solution, the fluorescence of SiNPs-ethanol was quenched significantly in the FRET process between negatively charged amino-capped SiNPs and positively charged citrate-stabilized AuNPs (Figure 3b), which suggests that AuNPs can be bound to SiNPs by the electrostatic interaction. On the other hand, the surface states of SiNPs can be influenced by AuNPs and they exhibited a strong effect on the fluorescence intensity of the SiNPs-ethanol solution. In the presence of L-Cys, AuNPs were able to be removed from the surface of SiNPs because of the stronger coordination ability of the thiols with AuNPs compared to that of the electrostatic interaction. Due to this effect, SiNPs-ethanol solution can be used to detect L-Cys. Therefore, the FRET efficiency between SiNPs and AuNPs was attenuated, resulting in the fluorescence recovery of the quenched SiNPs-ethanol solution (Figure 3c). Since the degree of attenuation on the FRET efficiency is strongly dependent on the amount of L-Cys, a highly sensitive assay for L-Cys detection was developed. Compared to some other complicated and costly biological methods for constructing an AuNP complex system [18,19,21], the FRET strategy here, based on electrostatic interaction, is simple and practical. The principle of this method, based on the decrease of the PL intensity of the SiNPs-ethanol solution, results in FRET assemblies between amino-capped SiNPs and citrate-stabilized AuNPs via electrostatic interaction.

**Figure 4:**
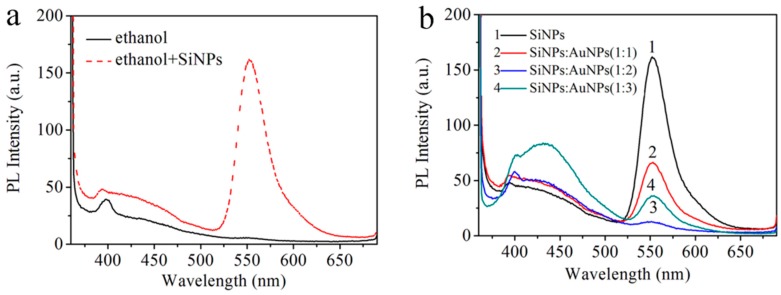
(**a**) Fluorescence emission spectra of ethanol and SiNPs-ethanol; (**b**) Fluorescence emission spectra of SiNPs and AuNPs at different volume ratios (*λ*_ex_: 350 nm, *λ*_em_: 550 nm).

Porous SiNPs in ethanol as a probe were efficiently synthesized and characterized by fluorescence spectra. In Figure 4a, there is almost no fluorescence peak of ethanol observed at around 550 nm with the excitation at 350 nm. In the presence of SiNPs, the significant fluorescence intensity which increased at 550 nm can be observed. In addition, other solvents such as buffer solution and DI water were also investigated. However, only the addition of ethanol resulted in the fluorescence enhancement at around 550 nm. We think that the obtained emission intensity maximum of SiNPs-ethanol solution at 550 nm originates from the C-C-O stretch, C-O stretch and symmetric CH_2_ stretch of ethanol, and porous SiNPs enhance the PL intensity of ethanol due to the quantum confinement effect of SiNPs. To realize the detection performance of L-Cys by the ensemble of SiNPs-ethanol and AuNPs and to obtain the response rate of the fluorescence signal of SiNPs-ethanol to AuNPs, the fluorescence spectra of SiNPs and AuNPs at different volume ratios were evaluated. In Figure 4b, after AuNPs were added into SiNPs-ethanol solution, the PL intensity of SiNPs-ethanol decreased drastically at 550 nm. The PL intensity was enhanced with a further increase of the volume ratio and reached its minimum when the volume ratio of the SiNPs and AuNPs mixture was 1:2. From the morphology of the AuNPs-SiNPs in Figure 1c,d, we think that AuNPs were absorbed on the surface of SiNPs, leading to the quenching of the fluorescence of the SiNPs-ethanol solution. To realize the detection performance of L-Cys with the combination of the SiNPs-ethanol solution and AuNPs, the assay conditions were carefully optimized. Consequently, the fluorescence intensity was quenched the most when the volume ratio of the SiNPs and AuNPs mixture was 1:2, which was chosen as the optimized condition.

**Figure 5:**
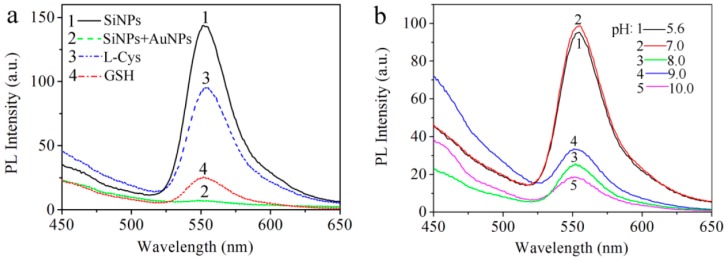
(**a**) Fluorescence emission spectra of SiNPs-AuNPs in ethanol (pH: 5.6) in the presence of 5 mM L-Cys and GSH. (1) SiNPs-ethanol solution, (2) SiNPs-AuNPs in ethanol, (3) SiNPs-AuNPs in ethanol in the presence of 5 mM L-Cys and (4) SiNPs-AuNPs in ethanol in the presence of 5 mM GSH; (**b**) The fluorescence intensity of SiNPs-AuNPs in ethanol in the presence of L-Cys under different pH (*λ*ex: 350 nm, *λ*em: 550 nm).

As shown in Figure 5a1, the as-prepared SiNPs-ethanol solution initially exhibited a strong characteristic fluorescence emission band at 550 nm. In Figure 5a2, the fluorescence of the SiNPs-ethanol solution was efficiently quenched by AuNPs when AuNPs were added into the SiNPs-ethanol solution. This indicates the possibility of energy transfer from SiNPs-ethanol to the AuNPs through the electrostatic interaction between SiNPs-ethanol and AuNPs. To turn back the fluorescence, we introduced L-Cys and the fluorescence of SiNPs-ethanol was successfully resumed. As shown in Figure 5a3, there was a significant enhancement in the fluorescence intensity only in the presence of L-Cys. The PL spectral responses of SiNPs-ethanol towards two amino acids, including L-Cys and glutathione (GSH), were investigated. It can be clearly seen from Figure 5a4 that the addition of thiol-contained GSH can result in fluorescence enhancement. From Figure 5a3,a4, we think that this method can detect thiol-contained species. Most importantly, the fluorescence signal of the system was lower than that of the system in the presence of L-Cys when other thiol-contained amino acids were added. In the following work, we selected the system of SiNPs-ethanol and AuNPs to study the recognition ability for L-Cys.

The pH value of a system is often considered as a significant influence factor on interactions. So the effect of the pH range on the interaction of L-Cys with AuNPs was also studied. It has already been reported that only at relatively low pH values does the amine group of amino acids bind with AuNPs, whereas the thiol group can bind with AuNPs at high pH values (pH > 5) [18]. In this experiment, the pH of the SiNPs-ethanol and AuNPs solution was found to be 5.6 and the other pH values of the solutions were adjusted to 7.0, 8.0, 9.0 and 10.0 with 1.0 M NaOH. As show in Figure 5b, the PL intensity of the L-Cys capping SiNPs-AuNPs in ethanol solution was higher when the pH values varied from 5.6 to 7.0 than when the pH values varied from 8.0 to 10.0. Therefore, the pH range of 5.6–7.0 is effective for this method and the pH value of 5.6 was used later for further study.

**Figure 6:**
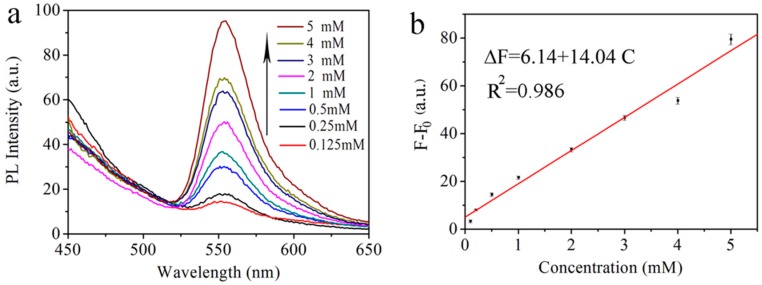
(**a**) Emission spectra of SiNPs-AuNPs in the presence of different L-Cys concentrations: 0.125, 0.25, 0.5, 1, 2, 3, 4 and 5 mM; (**b**) Linear relationship of L-Cys. The concentration of L-Cys is 0.125, 0.25, 0.5, 1, 2, 3, 4 or 5 mM, respectively.

In order to better understand the sensing process, the fluorescence intensity of SiNPs-AuNPs in ethanol was measured with different concentrations of L-Cys. As illustrated in Figure 6a, a recovering progress of the fluorescence intensity at 550 nm was observed with gradually increasing the concentration of L-Cys from 0.125 mM to 5 mM when the reaction mixture was excited at 350 nm. The observed emission intensity enhancement can be attributed to the surface states of SiNPs which will be influenced by AuNPs and exhibit a strong effect on the fluorescence intensity of the SiNPs-ethanol solution. We think L-Cys attach to the AuNPs and form Au-S bonds for the thiol groups, which prevent the fluorescence of SiNPs-ethanol from quenching by AuNPs. As the concentration of L-Cys in the solution became higher, more AuNPs were detached from the surface of SiNPs and there were less AuNPs left acting as effective quenchers in the system. These results are almost in agreement with the morphology of the AuNPs-SiNPs obtained from HR-TEM. Figure 6b shows the recovery of the fluorescence intensity ∆*F* (∆*F* = *F*_q_ − *F*_0_) corresponding to different L-Cys concentrations, where the recovery of the fluorescence signal corresponding to those concentrations was 3.2 (a.u.), 7.9 (a.u.), 14.4 (a.u.), 21.5 (a.u.), 33.2 (a.u.), 46.6 (a.u.), 53.7 (a.u.), and 79.4 (a.u.), respectively. The recovery of the fluorescence signal was found to be a linear function of the concentration of L-Cys in the range of 0.125 mM to 5 mM. The linear equation can be expressed as ∆*F* = 6.14 + 14.04*C* (*C* is the concentration of L-Cys) with the correlation coefficient R^2^ equal to 0.986. The detection limit (LOD) of L-Cys using this probe was calculated to be 35 μM (3σ/k, where σ is the standard deviation of a blank measurement and k is the slope of the linear equation). The relative standard deviation (RSD) for three independent repeated measurements indicates that the fluorescence response of SiNPs-AuNPs in ethanol toward L-Cys is highly repeatable. The linear fitness of the experimental data is given in Table 1.

The sensitivity of this porous silicon/ethanol probe to L-Cys is lower than some of the alternative approaches [11,25]. However, in the present study, we firstly fabricated a new method for the detection of L-Cys by using porous SiNPs in ethanol as a fluorescence “off-on” probe, which will broadly benefit the development of a new optical label-free biosensor based on porous nanoparticle materials. Porous SiNPs are nanostructured materials, and their sponge-like structure and large internal surface-to-volume ratio can reach several hundreds of square meters per cubic centimeter. This large value accounts for the enhanced reactivity of nanostructured materials with various adsorptive substances. The fluorescence can be restored after the addition of L-Cys because of the stronger coordination ability of the thiols with AuNPs via the electrostatic interaction, and L-Cys can release the adsorbed AuNPs from the surface of the SiNPs. However, there are still a few AuNPs that exist to be attached to SiNPs due to the sponge-like structure of the SiNPs, which will affect the sensitivity of the porous SiNPs-ethanol probe. Based on this work, we will try to explore new technology to improve the sensitivity of porous SiNPs which have a smaller size and a more uniform distribution.

## 4. Conclusions

In the present study, we firstly fabricated a new method for the detection of L-Cys by using porous SiNPs in ethanol as a fluorescence “off-on” probe. The excellent quenching efficiency between amino-capped porous SiNPs in ethanol and citrate-stabilized AuNPs via FRET provides an ideal “off-state”. Therefore, the fluorescence can be restored after the addition of L-Cys because of the stronger coordination ability of the thiols with AuNPs compared to that of electrostatic interaction, and L-Cys can release the adsorbed AuNPs from the surface of the SiNPs, which means the surface states of SiNPs can be influenced by AuNPs and they exhibit a strong effect on the fluorescence intensity of the SiNPs-ethanol solution. This method will broadly benefit the development of a new thiol biosensor based on porous nanostructured materials, and the proposed procedure is also expected to develop a variety of functional nanoparticles to form other novel kinds of nanosensors.

## Figures and Tables

**Table 1:** PL data of the SiNPs-AuNPs FRET biosensor to different concentrations of L-Cys in Figure 6. The ∆*F*_1_, ∆*F*_2_ and ∆*F*_3_ are fluorescence signal quenchings corresponding to three samples; $\bar{\Delta F}$ is the average value of the fluorescence signal quenching, *s* is the standard deviation and RSD is relative standard deviation, where $s=\sqrt{\frac{\sum_{i=1}^{n}{\left(\Delta F_{i}-\bar{\Delta F}\right)}^{2}}{n-1}}$, $RSD=\frac{s}{\bar{\Delta F}}\times 100\%$.

Concentrations	∆*F* (a.u.)			$\bar{\Delta F}$ (a.u.)	*s* (a.u.)	RSD	$\bar{\Delta F}$ ± *s* (a.u.)
	∆*F*_1_	∆*F*_2_	∆*F*_3_				
L-Cys (0.125 mM)	3.5	3.6	2.5	3.2	0.6	18.5%	3.2 ± 0.6
L-Cys (0.25 mM)	7.6	8.0	8.0	7.9	0.3	3.8%	7.9 ± 0.3
L-Cys (0.5 mM)	14.4	15.0	13.7	14.4	0.7	4.9%	14.4 ± 0.7
L-Cys (1.0 mM)	21.2	21	22.4	21.5	0.7	3.2%	21.5 ± 0.7
L-Cys (2.0 mM)	34.1	33.2	32.4	33.2	0.9	2.7%	33.2 ± 0.9
L-Cys (3.0 mM)	46.4	47.7	45.6	46.6	1.0	2.1%	46.6 ± 1.0
L-Cys (4.0 mM)	52.5	53.3	55.3	53.7	1.4	2.6%	53.7 ± 1.4
L-Cys (5.0 mM)	78.2	77.9	82.1	79.4	2.3	2.9%	79.4 ± 2.3
